# The Impact of Vegetation Structure on Shaping Urban Avian Communities in Chaoyang District Beijing, China

**DOI:** 10.3390/ani15152214

**Published:** 2025-07-28

**Authors:** Anees Ur Rahman, Kamran Ullah, Shumaila Batool, Rashid Rasool Rabbani Ismaili, Liping Yan

**Affiliations:** 1 School of Ecology and Nature Conservation, Beijing Forestry University, Beijing 100083, China; anis9003617@bjfu.edu.cn (A.U.R.); rashid.rri1998@gmail.com (R.R.R.I.); 2 Department of Biology, The University of Haripur, Haripur 22600, Pakistan; kamran.ullah@uoh.edu.pk; 3 Department of Zoology, Wildlife and Fisheries, University of Agriculture Faisalabad, Faisalabad 38000, Pakistan; shumailabatool262@gmail.com

**Keywords:** point count method, avian community, parks, PCA, vegetation

## Abstract

This study examines the impact of vegetation structure on bird species richness and diversity across four urban parks in Chaoyang District, Beijing. Throughout the year, using the Point Count Method (PCM), a total of 68 bird species and 4279 individual observations were recorded, with surveys conducted across all four seasons to capture seasonal variations. The parks with more complex vegetation, such as those with a higher tree canopy cover of species like poplars, ginkgo, and Chinese pines, exhibited higher bird species richness. For example, Olympic Forest Park, with its dense vegetation structure, hosted 42 species, whereas parks with less diverse vegetation supported fewer species. An analysis using PERMANOVA revealed that bird communities in the four parks were significantly different from each other (F = 2.76, *p* = 0.04075), and every comparison between parks showed significant differences as well (*p* < 0.001). Variations in the arrangement and level of disturbance within different plant communities likely cause such differences. Principal component analysis (PCA) identified tree canopy cover and shrub density as key drivers of bird diversity. These findings underscore the importance of preserving urban green spaces, particularly those with a diverse range of native tree species, to conserve biodiversity and mitigate the adverse effects of urbanisation. Effective vegetation management strategies can enhance avian habitats and provide ecological and cultural benefits in urban environments.

## Simple Summary

The proposed research observes the effect of vegetation structure on bird diversity in four parks located in the Chaoyang District of Beijing in a complete one-year cycle (April 2023–March 2024). Point Count Methods were used to conduct the bird surveys (to record 68 species and 4279 individuals) and quadrat sampling to cover the vegetation, discovering that parks with more complicated vegetation harbour much more affluent bird communities. The most species-rich environment (42 species) was Olympic Forest Park, featuring a dense native canopy of trees (55% cover, including poplars, ginkgo, and pines) and a high shrub density (40%). Conversely, parks with less complex foliage, such as the Wenyuhe River Park (35% tree coverage), had fewer species supported. Through principal component analysis, the two variables—tree canopy cover and shrub density were identified as the major drivers of avian diversity, explaining more than 71 per cent of the variation in the first component. The distinct bird communities between the parks were detected with PERMANOVA tests (Global F = 2.76, *p* = 0.04075), and all two-sample comparisons revealed significance (*p* < 0.001), indicating the impacts of park-specific environments, such as vegetation structure, levels of human disturbance, and microclimate. Most importantly, the presence of low human disturbance in parks such as Olympic Forest was associated with high levels of biodiversity. These findings suggest that retaining structurally diverse native vegetation, especially multi-layered canopies and dense shrubs, is crucial for enhancing avian habitats in urban parks and natural areas. This article proposes a customised park management approach that prioritises vegetation complexity in resisting urbanisation pressures and promoting urban ecological resilience in rapidly growing cities.

## 1. Introduction

Urban green spaces are vital for supporting avian biodiversity, which in turn offers numerous ecosystem services. These include providing habitats for diverse bird species, enhancing aesthetic and recreational experiences for city dwellers and contributing to pest control and pollination. Such benefits underscore the importance of preserving and enhancing green spaces in urban areas [1,2].

Studies have found that both local and landscape factors are essential in determining the occurrence of avian species diversity [3,4].

Along the urbanisation gradient, avian community structure varies based on the trade-off between resources and risks, with suburban areas playing a key role in supporting a higher diversity of bird species [5,6].

However, the majority of these studies that focused on the relationship between urban birds and environmental characteristics were conducted in urban parks and other relatively natural green patches; they neglected the vital role of residential communities (i.e., large urban apartment blocks that form a contiguous spatial whole), where a large proportion of an urban resident’s daily life is lived and where their primary interaction with the natural environment often occurs [7,8,9]. This is especially true in China and other developing countries with a rapidly growing urban population. If appropriately and collectively managed, the greening of such spaces could offset a significant part of urbanisation’s detrimental environmental effects [6]. Studies have suggested that an average green patch size explains most of the differences between avian communities residing in different habitat types and that the significant contribution of small greening projects is from connecting existing green spaces [9,10].

As the human population continues to grow, landscapes are increasingly affected by urbanisation. In 2010, more than 50% of the world’s population resided in urban areas, and by 2050, it is expected that even 70% of the human population will live in cities (UN 2012). This ongoing urban development leads to the fragmentation, isolation, and degradation of natural habitats, accompanied by severe impacts on the biotic communities living in urban environments, such as arthropods [11,12]. Numerous studies on urban bird communities have also shown that urbanisation can cause changes in community composition, a decrease in species richness, and a loss of species diversity [13,14,15,16,17]. The primary objectives of this study are to investigate the impact of various vegetation characteristics, including tree canopy cover, shrub density, and ground cover, on the richness and diversity of bird species in urban parks; to determine which vegetation features have the most substantial impact on bird community composition and distribution through PCA; and to offer urban park management strategies that prioritise vegetation structures supporting higher bird diversity based on this study’s findings.

## 2. Materials and Methods

### 2.1. Study Area

**Figure 1:**
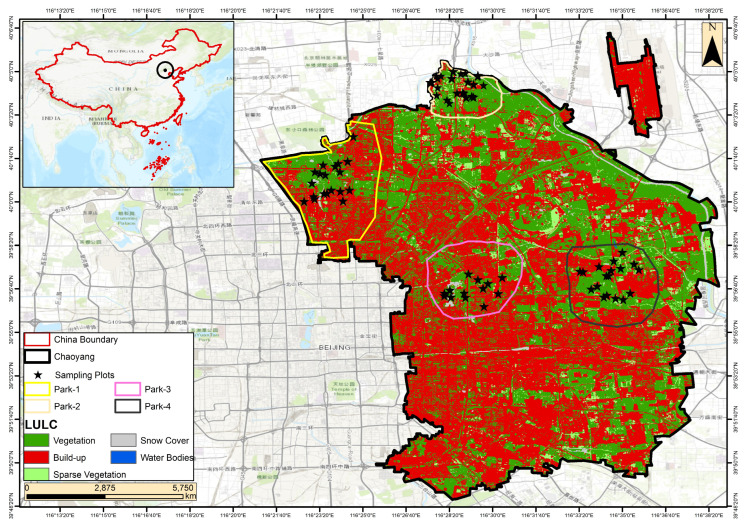
Map of the study area.

The spatial distribution of bird habitats in Chaoyang District is shown in Figure 1, highlighting key parks and their respective vegetation types. Chaoyang District is located at approximately 39°55′ N, 116°26′ E, with an elevation that generally ranges between 40 and 60 m, reflecting the flat topography characteristic of the North China Plain. This area covers about 478 km^2^. Chaoyang is a significant urban region that encompasses the Central Business District, numerous embassies, and several notable parks that contribute to its ecological and recreational landscape. In addition to green spaces, the district is traversed by several important water bodies, including the Liangma River, Tonghui River, and Bahe River, which serve as ecological corridors supporting both aquatic and terrestrial biodiversity. These rivers play a vital role in shaping microhabitats, maintaining local humidity, and offering critical resources for bird populations. Their integration into urban landscapes also enhances ecological connectivity between fragmented green patches. In Beijing’s Chaoyang District, approximately 60–70% of the territory is built up, with green spaces, including parks, accounting for 20–30%. The district’s green areas are home to a variety of vegetation, including trees such as poplars, ginkgo, and Chinese pines, shrubs such as Chinese privet and boxwood, and grasses such as Kentucky bluegrass and tall fescue, all of which contribute to the district’s balance of urban and natural beauty [18].

In this study, bird occurrence data were sourced from the Biodiversity Mapping website, initially with a spatial resolution of 10 × 10 km^2^. With ArcGIS software (version 10.5), the data were first loaded and low-pass-filtered. Subsequently, spatial resampling techniques were applied to achieve a reduced resolution of 1 km^2^. The classification of bird occurrence into three distinct levels (Medium and High) was carried out based on population thresholds, providing a nuanced perspective on avian habitats across the resampled grid. The utilisation of ArcGIS tools facilitated a comprehensive analysis, allowing for in-depth spatial exploration and visualisation. This methodology enhances our understanding of bird ecology and biodiversity patterns within larger geographic contexts, offering valuable insights into the relationships between bird populations and their environments [19].

### 2.2. Birds Survey

The bird survey was conducted over one year, from April 2023 to March 2024, using the Point Count Method (PCM) across four parks in Chaoyang District, Beijing. This method utilised 80 plots, with each park containing 20 plots measuring 20 m by 20 m. The survey was designed to cover all four seasons, spring, summer, autumn, and winter, ensuring seasonal variations in bird activity were accounted for. Sampling points were spaced 150 m to 200 m apart, with a 50 m buffer around each point count station to avoid spatial duplication. Observations were made using binoculars and guidebooks, with each session at a point count station lasting 15 min, during which all bird activity within the surrounding area was recorded. This survey collected detailed information on four bird communities, including species presence and encounter numbers, and based on these parameters, species richness and diversity were calculated [20].

### 2.3. Vegetation Survey

The vegetation survey was conducted concurrently with the bird survey using the Quadrat Method, focusing on different scales: 10 m × 10 m for trees, 5 m × 5 m for shrubs, and 1 m × 1 m for grasses, herbs, and bushes. A total of 80 quadrat plots were surveyed: 20 for trees, 20 for shrubs, 20 for herb and bush communities, and 20 for grasses. Each plant within the plots was meticulously identified and counted, providing comprehensive data on species richness, height, canopy cover, and ground cover. These detailed vegetation data are essential for understanding plant community structure and its relationship with bird species preferences and avian community dynamics. The methods employed adhered to established guidelines to maintain the reliability and comparability of the data, contributing to a robust analysis of the interactions between vegetation and bird communities [19].

### 2.4. Timing of Observations and Surveys

The field observations and floristic surveys were conducted over a period of one year, from February 2023 to March 2024. Bird counts were conducted regularly throughout this period to capture seasonal variations in bird abundance and behaviour. The periods were from 7:00 to 10:00 a.m. and from 3:00 to 4:00 p.m. Additionally, floristic surveys were conducted in parallel with the bird surveys to determine vegetation composition and structure. The surveys were not conducted only once but were repeated several times to ensure comprehensive coverage of the study area and to account for temporal variability in bird populations and vegetation dynamics.

### 2.5. Methods of Bird Species Determination

Bird species were identified through visual observation. Visual identification was supplemented with audio recordings to confirm the presence of particular species, especially those with distinctive vocalisations. This approach ensured accurate species identification and enhanced the reliability of the collected data. Climatological parameters and meteorological variables, including temperature and relative humidity, were recorded using data loggers strategically placed within the study area. These data loggers were programmed to capture environmental conditions at regular intervals throughout the study period, ensuring comprehensive coverage of climatic variations. Detailed information regarding the timing and methodology of data collection for climatological parameters will be explicitly outlined in the Materials and Methods section. The recording of climatological parameters, in addition to temperature and relative humidity, also included wind speed, which was recorded using data loggers strategically placed within the study area. These data loggers were programmed to capture wind speed measurements at regular intervals concurrent with the recording of temperature and humidity. The inclusion of wind speed data aims to provide a more comprehensive understanding of microclimate variability and its potential influence on bird species occurrence.

### 2.6. Statistical Method

The data obtained from bird surveys and vegetation assessments were subjected to rigorous statistical analysis to elucidate key patterns and relationships. Descriptive statistics, including mean, standard deviation, and frequency distributions, were calculated to characterise bird abundance and vegetation composition. Additionally, inferential statistical methods, such as correlation analysis and regression modelling, were employed to investigate the associations between bird occurrence and habitat characteristics. All statistical analyses were conducted using R software (version 4.3.2) with significance levels set at α = 0.05 unless otherwise stated.

### 2.7. Data Analysis

In R, principal component analysis was performed in XLSTAT 2014 software. Principal component analysis (PCA): We employed principal component analysis (PCA) to evaluate vegetation composition variables for birds and their habitats in urban parks [21].

## 3. Results

This study presents the findings from the analysis conducted to investigate the relationship between vegetation structure and bird species richness in urban parks of Chaoyang District, Beijing. The results are organised based on the research objectives, focusing on principal component analysis (PCA), biplot analysis, species richness, and seasonal variations.

A PERMANOVA test was performed to determine if the types of birds in the four urban parks across Chaoyang District differed. The test across all parks showed that the bird communities differed among the parks (F = 2.76, *p* = 0.04075, ***), indicating that park identity did play a role.

The results of the vegetation surveys across the four parks showed notable variation in plant community composition, canopy cover, and vegetation density.

Perma Anova compared individual sites.

To study which pairs of parks had different bird species, pairwise PERMANOVA analyses were run (see Table 1 for the results). Every comparison of park pairs yielded a significant result (*p* < 0.001), indicating that each park is home to its unique bird population.

In Table 2, all park pairs was clearly distinguished ecologically, as the species living in each are unique. The R^2^ values of 0.13–0.15 contribute to the evidence that park identity influences bird composition.

### 3.1. Principal Component Analysis

The eigenvalues for the principal components and their explained variances are summarised in Table 2. Principal component analysis (PCA) was performed to reduce the dataset’s dimensionality and identify the key vegetation variables influencing bird species richness across parks.

### 3.2. Biplot Analysis

**Figure 2:**
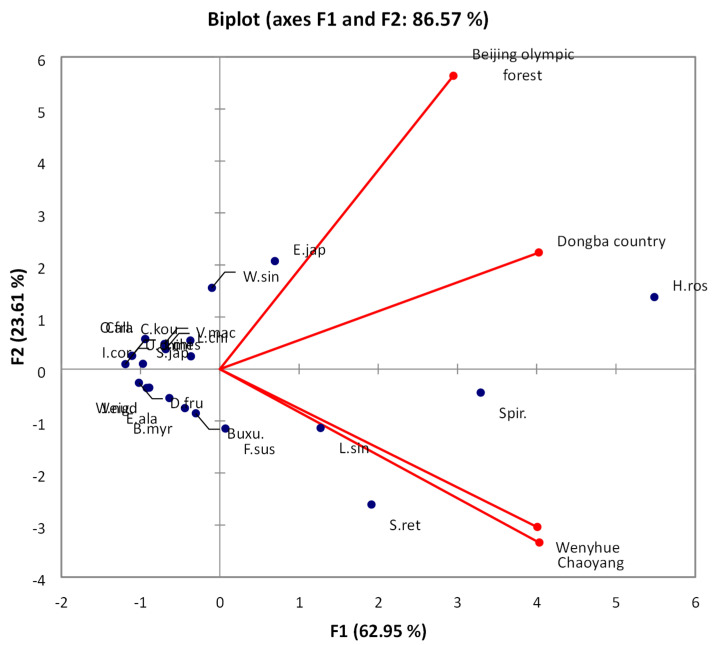
Pilot biplot of component loadings and scores for park dataset.

The biplot in Figure 2 illustrates the distribution of bird species and parks based on the first two principal components. A biplot was created to visualise the relationships between parks and bird species in terms of the first two principal components (F1 and F2).

In this Figure 2 depicts a biplot, a graphical representation that displays the samples’ scores on the principal components (PCs) and the loadings of variables on these PCs. The biplot displays the first two principal components, F1 and F2, which collectively explain 87.24% of the data’s variability. The F1 axis, denoted on the *x*-axis, accounts for 71.36% of the variability, while the F2 axis, denoted on the *y*-axis, accounts for 15.88%. The points representing Beijing Olympic Park, Chaoyang Park, Dongba Country Park, and Wenyuhe River Park are plotted based on their scores on these two primary components. The relative placements of the parks indicate variations and commonalities based on the underlying factors. *Zosterops oyspakensis* (Oriental White-eye) and *Ficedula aruensis* (Aruru Flycatcher) indicate the direction of the highest variance for those variables. The magnitude and orientation of these vectors reveal the degree of correlation between each species and the primary components, as well as the interrelationships between them. Species that are closely located in a particular park exhibit a more robust correlation.

Detailed species richness and associated vegetation characteristics for each park are listed in Table 3. Olympic Forest Park exhibited the highest bird species richness (42 species), followed by Chaoyang Park (35 species). The greater species richness in these parks can be attributed to their more diverse and dense vegetation structures, which provide suitable habitats for a wide range of bird species. Parks with higher tree canopy and shrub density were associated with more affluent bird communities, emphasising the role of complex vegetation in promoting biodiversity. Furthermore, parks with lower levels of human disturbance, such as Olympic Forest Park, tended to have higher species richness, suggesting that minimising human activity can contribute to better habitat conditions for birds.

Table 3 Summary of bird species richness, vegetation structure (tree canopy cover, shrub density, ground cover), average temperature, and human disturbance level for each of the four urban parks surveyed in Chaoyang District, Beijing.

### 3.3. Bird Species Richness

**Figure 3:**
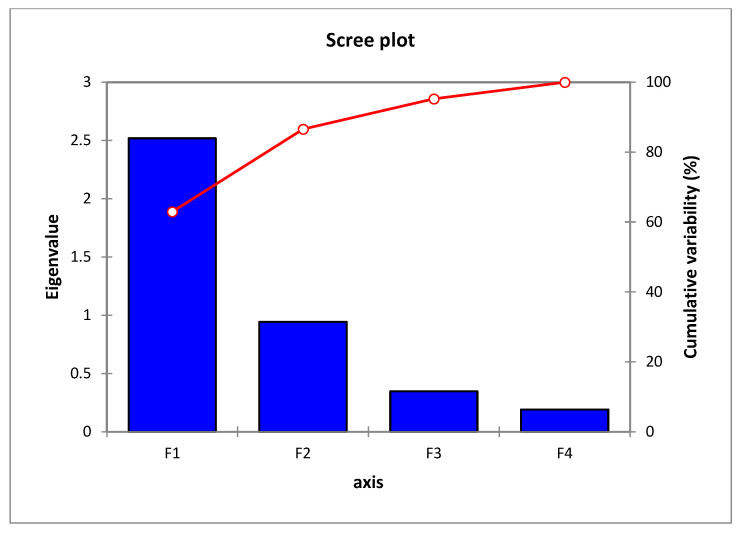
Scree plot displaying eigenvalues and cumulative variability for principal components.

The scree plot for the second PCA is shown in Figure 3.

A second principal component analysis (PCA) was conducted specifically for species richness variables, with the results displayed in Table 4.

Table 4 shows the eigenvalues obtained from the PCA, along with the percentage of variability explained by each principal component (F1, F2, F3, F4) and the cumulative percentage of variability explained.

**Figure 4:**
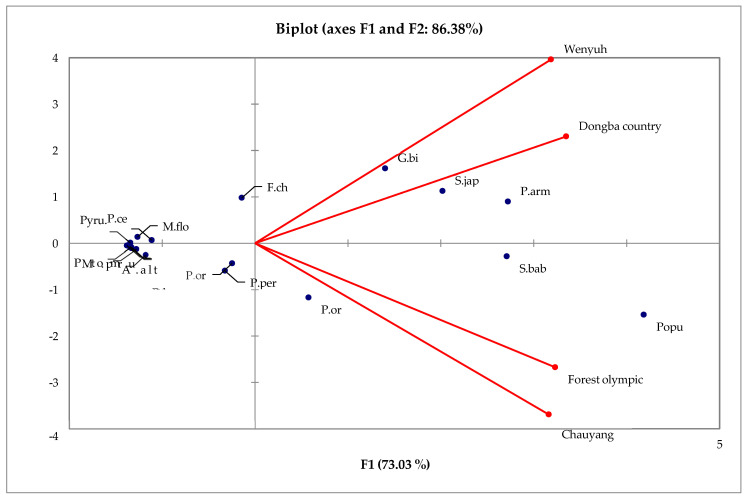
Biplot of the first two principal components from multivariate data analysis.

The scree plot displays the eigenvalues associated with each main component (PC) on the horizontal axis, labelled explicitly as F1, F2, F3, and F4. The eigenvalues indicate the extent of variation captured by each principal component (PC), and the height of the bars visually represents this variation. The initial bar (F1) exhibits the most significant height, indicating that this major component explains the most variability in the dataset. The red line, accompanied by markers, represents the cumulative percentage of variability accounted for by the successive inclusion of principal components. The value begins at around 60% at F1 and gradually increases to 100% by F4. The elbow, or the point where the red line begins to plateau (just following F1 in this graph), indicates the ideal number of primary components to consider. The plot is a regularly employed tool in principal component analysis (PCA) to determine the optimal number of principal components for subsequent investigation. The biplots in Figure 4 present the multivariate relationships between bird species and vegetation factors across parks.

This Figure 4 biplot displays the scores of different items (likely species or variables, represented by blue markers) and the loading of various factors (perhaps environmental variables or park names, represented by red vectors) on the first two principal components (F1 and F2). Variable F1 explains 62.95% of the overall variation, while F2 explains an additional 23.61%. When these two variables are combined, they account for 86.57% of the variance. The closeness of the blue markers to each other signifies the similarity of their profiles across all variables examined in the analysis. The vectors indicate the direction of the most significant rise in the respective variable. Therefore, the longer the vector, the more pronounced the influence of that variable on the distribution of the markers. Within this framework, it is evident that the “Beijing Olympic Forest” and “Chaoyang” exhibit a significant favourable influence on F1, while “Wenyhue” has a notable negative impact on F2, and “Dongba Country” has a minimal beneficial effect on F2. This implies that the first two principal components play a significant role in explaining the variation and that the other factors (such as parks) contribute to this variation in unique ways.

These fundamental components process important differences in habitat in an ecological sense among the four parks. The first component (F1, 73.03%) primarily represents the variations in tree cover and human disturbance Figure 4, whereas the second component (F2, 13.34%) is associated with shrub density and ground cover. Olympic Forest Park and Chaoyang Park are heavily loaded with F1, which is related to more canopy cover and less disturbance, supporting woodland species. On the other hand, Wenyuhe River Park and Dongba Country Park have greater F2 values, probably because their shrubby density and more open or riparian lands contribute to the presence of shrubland and wetland bird species. This proves that the parks possess dissimilar habitat structures, which determine their respective bird communities.

Table 5 Species richness (S), total number count (N) and diversity measure such as ShannonWiener measure of diversity index (H o), Simpson measure of dominance index (D), inverse Simpson index (InvD), Pieloux evenness measure of diversity index (J), Margalef and Menhinick measure of diversity index(richness = A − B/C and richness = A/B − C) and Fischers alpha measure of diversity split across the four urban parks and pooled habitat types surveyed within Chaoyang District of Beijing. Values indicate seasonal surveys that are pooled.

## 4. Discussion

This study demonstrates that vegetation structure has significant influence on bird biodiversity in urban settings. The results from the principal component analysis (PCA) indicate that tree canopy cover and shrub density are key determinants of bird species richness in Chaoyang’s urban parks, consistent with previous research. The eigenvalue analysis reveals that the first principal component (F1), which accounts for over 70% of the variability, is significantly influenced by these vegetation variables, underscoring their importance in supporting diverse bird communities. In addition to vegetation structure, spatial variation among parks was found to significantly influence the composition of avian communities. Results from the PERMANOVA test indicate significant differences in bird assemblages among the four studied parks (F = 2.76, *p* = 0.04075), confirming that the identity of each park influences the way bird communities differ from one another. Additional PERMANOVA testing between pairs of parks revealed that all parks were statistically different (*p* < 0.001) and had high R^2^ values, which supports the notion that each park has its unique bird community. The differences could result from the way parks are vegetated, the human activities in them, and variations in their nearby climate. Olympic Forest Park had more species than any other park in the area. These parks were characterised by highly diverse vegetation and were also the least disturbed by humans. The results support previous research indicating that the arrangement of spaces in an urban area can influence the types of species found there [10,17].

This corresponds with the findings of Lerman (2011) [6], who highlighted the conservation significance of residential yards and their contribution to avian biodiversity.

Species richness was highest in Olympic Forest Park, which exhibited the most complex vegetation structure, with a tree canopy cover of 55% and a shrub density of 40%. In contrast, parks with less diverse or sparser vegetation, such as Wenyuhe River Park, supported fewer bird species, illustrating the positive correlation between vegetation complexity and bird diversity. These results align with those of Strohbach et al. (2013) [10], who found that green spaces with more diverse vegetation enhance bird diversity, particularly in urban areas where natural habitats are fragmented.

Furthermore, this study noted that parks exhibiting reduced human disturbance, such as Olympic Forest Park, correlated with increased species richness, substantiating the premise that diminishing human activity can enhance habitat quality for avifauna.

This finding aligns with McKinney (2006) [22], who reported that urbanisation leads to biotic homogenisation. However, well-managed green spaces can mitigate some of these effects by providing refuge for diverse bird species.

The seasonal variations in bird species richness, as captured through year-long surveys, further highlight the dynamic relationship between vegetation and bird communities. Vegetation not only provides habitat but also influences resource availability, which fluctuates seasonally, affecting bird presence and behaviour [19]. The combination of year-round data collection and detailed vegetation analysis offers robust insights into how different types of vegetation support bird communities throughout the year.

A stronger vertical vegetation structure supports higher bird species richness by creating diverse foraging and nesting niches at different heights. Canopy layers provide refuge from human disturbance—especially for species sensitive to ground-level activity such as dog walking or foot traffic—while ensuring adequate flight distance. This vertical separation enables the coexistence of species and reduces competition. As shown by Zaplata and Dullau (2022) [23], even small urban green spaces can support distinct bird communities through spatial and temporal partitioning driven by vegetation complexity. Seasonal differences in avifauna across the studied parks also suggest that structurally diverse habitats provide more stable resources throughout the year, benefiting both resident and migratory species.

## 5. Conclusions

In our one-year study across four urban parks in Chaoyang District, Beijing, we observed clear seasonal and spatial variation in bird diversity. Parks with more complex vegetation structures characterised by high canopy cover, dense shrubs, and diverse ground cover supported consistently richer avian communities. This highlights the ecological significance of vertical vegetation structure in supporting bird diversity within urban landscapes. The results highlight the need for urban planners to prioritise vegetation management in parks to foster biodiversity, reduce human disturbance, and enhance ecological benefits in rapidly urbanising areas.

## Figures and Tables

**Table 1:** A PERMANOVA test.

Df	Sum Sq	Mean Sq	F Value	Pr (>F)
3	65.31232	21.77077	2.758496	0.04075 ***
5436	42902.33	7.892261		

Note: *** indicates *p* < 0.05.

**Table 2:** Comparison in different groups.

Comparison	R2	F_Value	*p*_Value
(1) Beijing Olympic Park vs. Chaoyang Park	0.15282	4.5697	0.0001 ***
(2) Beijing Olympic Park vs. Dongba Country Park	0.13456	3.7812	0.0003 ***
(3) Beijing Olympic Park vs. Wenyuhe River Park	0.14321	4.1235	0.0001 ***
(4) Chaoyang Park vs. Dongba Country Park	0.14532	3.9123	0.0005 ***
(5) Chaoyang Park vs. Wenyuhe River Park	0.13987	4.0012	0.0002 ***
(6) Dongba Country Park vs. Wenyuhe River Park	0.15098	4.2156	0.0001 ***

Note: *** indicates *p* < 0.05.

**Table 3:** Bird species richness and vegetation characteristics across parks.

Park	Area in Hectares	Species Richness	Tree Canopy Cover (%)	Shrub Density (%)	Ground Cover (%)	Average Temperature (°C)	Human Disturbance Level
Chaoyang Park	680	35	45	30	70	22.5	Medium
Dongba Country Park	288	27	50	25	60	21	High
Olympic Forest Park	470	42	55	40	80	23	Low
Wenyuhe River Park	320	30	35	45	65	20.5	Medium

**Table 4:** Eigenvalues: These are the eigenvalues obtained from the principal component analysis (PCA).

PCA	F1	F2	F3	F4
Eigenvalue	2.855	0.635	0.328	0.182
Variability (%)	71.364	15.881	8.194	4.562
Cumulative %	71.364	87.245	95.438	100

**Table 5:** The total bird species richness for each park is presented in the table.

Site	S	N	H	D	InvD	J	Margalef	Menhinick	Fisher_Alpha
Chaoyang	56	1093	3.091819	0.925453	13.41443	0.768087	7.860869	1.693862	12.4917062
Dongba country	56	1060	2.89646	0.904858	10.51056	0.719555	7.895465	1.720026	12.60111705
Forest Olympic	59	1202	2.981406	0.910482	11.17093	0.731178	8.178526	1.701766	13.00335998
Wenyuhe river	61	1051	3.29843	0.937915	16.10699	0.802367	8.623791	1.881605	14.10674452
Grassland	62	1207	3.056978	0.912088	11.37497	0.740702	8.596522	1.784588	13.84016678
Urban Forest	59	176	3.651677	0.961067	25.68491	0.89556	11.21752	4.447292	31.13307409
Wetland	65	1469	3.11412	0.92074	12.61663	0.746007	8.776336	1.695909	13.92417486
Woodland	68	1533	3.05482	0.917155	12.07074	0.723975	9.13431	1.736752	14.57654866

## Data Availability

The original contributions presented in this study are included in the article. Further inquiries can be directed to the corresponding author.

## Abbreviations

**Abbreviation**	**Scientific Name**	**Common Name**
*Z. oys*	*Zosterops oys*	Oriental White-eye
*F. aru*	*Ficedula aruruensis*	Aruru Flycatcher
*E. jap*	*Egretta japonica*	Little Egret
*S. ret*	*Sitta reticulata*	Reticulated Nuthatch
*P. mon*	*Parus monticolus*	Green-backed Tit
*P. major*	*Parus major*	Great Tit
*T. mer*	*Turdus merula*	Common Blackbird
*C. fam*	*Cyanistes familaris*	Eurasian Blue Tit
*G. gland*	*Garrulus glandarius*	Eurasian Jay
*D. maj*	*Dendrocopos major*	Great Spotted Woodpecker
*S. vari*	*Sitta varia*	Varied Nuthatch
*P. pica*	*Pica pica*	Eurasian Magpie
